# Structuring of ZnTiO_3_/TiO_2_ Adsorbents for the Removal of Methylene Blue, Using Zeolite Precursor Clays as Natural Additives

**DOI:** 10.3390/nano11040898

**Published:** 2021-04-01

**Authors:** Ximena Jaramillo-Fierro, Silvia González, Fernando Montesdeoca-Mendoza, Francesc Medina

**Affiliations:** 1 Departamento de Química y Ciencias Exactas, Universidad Técnica Particular de Loja, San Cayetano Alto, Loja 11-01-608, Ecuador; sgonzalez@utpl.edu.ec (S.G.); famontesdeoca2@utpl.edu.ec (F.M.-M.); 2 Departamento d’Enginyería Química, Universitat Rovira i Virgili, Av Països Catalans 26, 43007 Tarragona, Spain; francesc.medina@urv.cat

**Keywords:** ZnTiO_3_/TiO_2_, zeolite, clay, adsorption, methylene blue

## Abstract

Adsorption is an effective method of removing harmful pollutants from air and water. In the present study, zeolites prepared by sol-gel method from two Ecuadorian clays were combined with precursor clays and the ZnTiO_3_/TiO_2_ semiconductor for adsorbing methylene blue (MB) as a water contaminant. The synthesized compounds were characterized using powder X-ray diffraction, X-ray fluorescence, scanning electron microscopy, energy dispersive X-ray, and surface area measurement. These compounds were combined to form cylindrical extrudates of 0.2 cm (diameter) and 1.0 cm (length). The adsorption characteristics of the composites were measured using batch sorption studies as a function of pH, initial concentration, and contact time. The pseudo-second-order model and the Langmuir isotherm model were better suited to the adsorption process. The equilibrium state was achieved around 180 min of adsorption, and a pH of 7 was established as the optimal operating condition. The maximum adsorption values of the dye were obtained with the composites derived from G-Clay, whose average adsorption capacity was 46.36 mg g^−1^, in contrast with composites derived from R-Clay, whose average adsorption value was 36.24 mg g^−1^. The results reflect that synthesized composites could be used potentially for the removal of cationic dye from wastewater.

## 1. Introduction

Dyes are substances with an important application in various industries such as food, printing, plastics, textiles, paper, leather, and pharmaceutical products [1]. Methylene blue (MB) dye, which is widely used in the textile industry, is one of the most famous water pollutants [2]. MB is frequently discharged into the environment without restriction or control, having dangerous effects on living organisms [3]. MB also affects the environment and damages the ecosystem’s balance since, even in low concentrations, the presence of this dye in water inhibits photosynthesis in the aquatic environment due to the reduction of solar irradiation [4,5]. Therefore, effluent treatment with MB dye prior to its discharge is of interest due to its harmful impacts on receiving water [6].

Numerous technologies, including biological degradation as well as chemical and physical methods, have been extensively studied to treat wastewater contaminated with dyes [7,8,9,10,11,12]. Adsorption is an attractive method for removing pollutants from effluents as it is a flexible process in terms of design and operation and produces pollutant-free effluents that are suitable for reuse [13,14]. The efficiency of the adsorption process depends on the characteristics of the solution medium, the adsorbate, and the adsorbent [15,16]. An ideal adsorbent should have a porous structure, a large specific surface area, good physical, mechanical and chemical stability, and high affinity for the adsorbate [17]; therefore, research on materials that are both efficient and economical should be encouraged.

In recent years, there has been a growing interest in the use of metallic and non-metallic oxides to adsorb organic and inorganic contaminants. ZnTiO_3_ is a mixed oxide obtained by the sol-gel method during the synthesis of coupled ZnO-TiO_2_ [18,19,20]. This oxide is usually synthesized with some impurity phases such as anatase and rutile [21,22,23]. This mixed oxide is interesting for its technological applications as an adsorbent and photocatalyst; it is also a low-cost and environmentally friendly material [24,25]. Previous studies show that ZnTiO_3_/TiO_2_ can eliminate methylene blue dye from aqueous systems, being of interest for effluent treatment [26].

Other interesting adsorbent materials are clays since they are abundant in nature, are low-cost, and have high absorption properties and ion exchange potential. Most clays are also suitable precursors for the synthesis of materials with improved properties, due to their mineralogical composition [27]. Several authors have reported the use of clays to obtain zeolites with greater structural regularity and greater porosity, as well as a particular surface chemistry that improves their adsorbent capacity [1]. In recent years, zeolite FAU and zeolite LTA have shown important applications as catalysts, ion exchange materials, and adsorbents. Both zeolites can be synthesized from natural solids using the hydrothermal method, making them suitable high value-added materials to efficiently remove pollutants from wastewater.

Although the literature indicates that various adsorbents can be widely used for wastewater treatment, their recovery at the end of the process limits their practical application. Structured materials offer an alternative to avoid the separation step at the end of the process [28,29,30]. The structured materials are prepared in the presence of a binder or matrix. The literature shows several types of binders for the preparation of structured materials, for instance: clays, titania, alumina, and/or silica or combinations of these materials [31,32]. Clays represent a very promising alternative because of their high mechanical and chemical stability [33,34]. Clays are attractive minerals for use in adsorption, catalysis processes, controlled release of active compounds and also offer an interesting route to revalue local natural resources [35,36,37].

Clays, being inexpensive and widely available, represent an attractive binder for the immobilization of a variety of active compounds that are in powder form [38]. The clays used as inorganic binders allow to achieve mechanically resistant structured bodies after the respective calcination process. This thermally induced increase in mechanical strength is probably related to the creation of new and stronger bonds between the constituent powders that also improve the wear resistance of the material during the process [39].

Clay minerals are an important source of raw material for obtaining, through physical and chemical modification, new supports with desired porosity, appropriate inorganic structure and adequate SiO_2_/Al_2_O_3_ ratios, providing solids with unique characteristics [39]. Therefore, clays represent a promising material for cleaning natural resources and improving the quality of life of the population [40].

Several authors have studied the adsorption of methylene blue from aqueous systems using clays [41,42,43,44,45,46] and zeolites [47,48,49,50,51,52] as adsorbents. In addition, we previously reported the use of ZnTiO_3_/TiO_2_ to remove methylene blue. The results of this study showed that this mixed oxide can degrade the dye, especially by photocatalysis, although it also showed slight adsorbent activity [26]. However, no studies have been found in the literature in which ZnTiO_3_/TiO_2_, zeolites, and clays are combined to prepare composites that serve to adsorb colorants in general, and methylene blue in particular, from aqueous systems. Additionally, despite the great industrial importance of producing structured adsorbents from porous powders, few papers in the academic literature are devoted to reporting studies of the structuring [39].

In light of the above, this work reports the synthesis of ZnTiO_3_/TiO_2_ and zeolites from natural clays, as well as the preparation of extruded composites from these materials for the removal of methylene blue in aqueous effluents. The synthesized compounds were characterized by X-ray diffraction (XRD), X-ray fluorescence (XRF), scanning electron microscopy (SEM-EDX), and specific surface area (BET). In addition, the adsorption capacity of the synthesized extruded composites was measured in batch experiments, varying the pH of the solution, the concentration of the adsorbent, and the contact time.

## 2. Materials and Methods

### 2.1. Materials

All of the reagents used in this work were of analytical grade and used without additional purification: Ti(OC_3_H_7_)_4_ (Sigma Aldrich, St. Louis, MO, USA, 98%), CH_3_COOH (Fisher Scientific, Waltham, MA, USA, 99.8%), CH_3_OH (Sigma Aldrich, St. Louis, MO, USA, ≥99.8%), HCl (Fisher Scientific, Waltham, MA, USA, 37%), cetyl-trimethyl ammonium chloride (C_19_H_42_NCl) (Sigma Aldrich, St. Louis, MO, USA, 25%), H_2_O_2_ (Sigma Aldrich, St. Louis, MO, USA, 35%), AgNO_3_ (Sigma Aldrich, St. Louis, MO, USA, >99.8%), HNO_3_ (Sigma Aldrich, St. Louis, MO, USA, 69%), Zn(CH_3_COO)_2_·2H_2_O (ACS, St. Louis, MO, USA, ≥98%), C_16_H_18_ClN_3_S·xH_2_O (Sigma Aldrich, St. Louis, MO, USA, ≥95%), NaOH (ACS, St. Louis, MO, USA, ≥97%).

### 2.2. Clay Purification

The raw clays were collected from southern Ecuador. The clays were labeled R-Clay and G-Clay due to their red and gray color, respectively. The clay samples were ground and sieved to 200-mesh (0.074 mm) size. Carbonates of calcium and magnesium were removed using hydrochloric acid (0.1 N) at a ratio of 10 mL g^–1^. The organic matter present in the clay samples was oxidized by the addition of H_2_O_2_ (33%) at a ratio of 10 mL g^−1^ under agitation for 2 h at room temperature. After centrifugation, the purified clays were washed with distilled water for the removal of Cl^−^ ions; this was checked with a test with AgNO_3_. The clay adsorption sites were activated with nitric acid (0.8 N) in a proportion of 10 mL g^−1^. The activated clay samples were centrifuged, washed with distilled water, and dried at 60 °C for 24 h.

### 2.3. Synthesis of the ZnTiO_3_/TiO_2_ Semiconductor

The ZnTiO_3_/TiO_2_ compound was synthesized following a modified sol-gel method described by other authors [53,54]. A quantity of TiIPO in iPrOH (70 *v*/*v*%) was dispersed at room temperature. An aqueous solution formed by Zn(acet)/water/iPrOH was slowly added, using ZnO/TiO_2_ at 1:3 molar ratio. The amount of water was stoichiometric for hydrolyzing the TiIPO molecules, adding a solution containing a 50 *v*/*v*% iPrOH/water ratio. The synthesis was performed at room temperature. The reaction system was additionally stirred for 30 min. The mixture was kept under stirring at room temperature for another 30 min after the formation of a precipitate. The precipitate was dried at 60 °C for 24 h and calcined at 500 °C for 4 h. Finally, the products were cooled at room temperature.

### 2.4. Synthesis of Zeolite from Ecuadorian Clays

The synthesis conditions were set based on previous work on zeolite synthesis from aluminosilicate gels [55,56]. The preliminary process consisted of the following steps: 20 g of each clay were mixed with 25 g of NaOH and dissolved in 50 mL of water, to form a homogeneous mud. Each clay-NaOH mud was calcined at 800 °C for 5 h.

To synthesize zeolite from R-Clay, the necessary reagents were added until reaching the composition established for the synthesis mixture: SiO_2_/Al_2_O_3_ = 2.47, Na_2_O/SiO_2_ = 3.0, H_2_O/Na_2_O = 20. The calcined product was crushed and suspended in 125 mL of water. The mixture was stirred and homogenized at room temperature for 1 h. Hydrothermal treatment of the mixture was carried out in covered containers and heated to 90 °C for 2 h. Finally, the solid product was filtered, washed with water to remove excess alkali, and dried at 90 °C overnight. The main product obtained with this procedure was labeled R-Zeolite and identified as Na-LTA type zeolite with small amounts of Na-FAU type zeolite. To synthesize zeolite from G-Clay, the necessary reagents were added to achieve the composition established for the synthesis mixture: SiO_2_/Al_2_O_3_ = 4.0, Na_2_O/SiO_2_ = 1.65, H_2_O/Na_2_O = 40. The calcined product was crushed and suspended in 128 mL of water. The mixture was stirred at room temperature for 1 h, to homogenize. Subsequently, the solution was aged for 24 h at room temperature. After this, hydrothermal treatment of the mixture was carried out in covered containers and heated to 90 °C for 24 h. Finally, the solid product was filtered, washed with water to remove excess alkali, and dried at 90 °C overnight. The main product obtained with this procedure was labeled G-Zeolite and consisted of Na-FAU type zeolite with small amounts of Na-P1 type zeolite.

### 2.5. Preparation of Extruded Composites

For the evaluation of solid materials, cylindrical extrudates with approximate dimensions of 1.0 cm in length and 0.2 cm in diameter were prepared. The preparation of these solids was performed by mixing the ZnTiO_3_/TiO_2_ semiconductor with zeolite and the precursor clay at a ratio of 30:30:40, respectively. Extrudates were also prepared by mixing the ZnTiO_3_/TiO_2_ semiconductor with clay at a ratio of 60:40, respectively. Finally, clay extrudates were also prepared by the same process using only clay in the composition without adding any other components. An amount of water (approximately 35%) was added to each mixture to form a paste with good plasticity. These mixtures were extruded with a 2.5 mm diameter syringe. The extrudates were dried at 90 °C for 2 h and finally calcined at 500 °C for 8 h. The presence of clay is important for the formation of extrudates. In this study, extrudates prepared only with semiconductor and zeolite were not considered because they disintegrate easily. The extruded composites prepared were labeled as follows: R (R-Clay), RT (R-Clay and ZnTiO_3_/TiO_2_), RZT (R-Clay, R-Zeolite, and ZnTiO_3_/TiO_2_), G (G-Clay), GT (G-Clay and ZnTiO_3_/TiO_2_), GZT (G-Clay, G-Zeolite, and ZnTiO_3_/TiO_2_).

### 2.6. Characterization

The synthesized materials were characterized using a JEOL JSM 6400 scanning electron microscope (SEM-EDX) (JEOL, Peabody, MA, USA). X-ray fluorescence (XRF) measurements were recorded in a Bruker S1 Turbo SDR portable spectrometer (Bruker Handheld LLC, Kennewick, WA, USA), using the Mining Light Elements measurement method. The X-ray diffraction (XRD) measurements were recorded in a Bruker AXS D8-Discover diffractometer (Bruker AXS, Karlsruhe, Germany) equipped with a vertical θ-θ goniometer, parallel incident beam (Göbel mirror), and a HI-STAR General AREA Diffraction Detection System (GADDS) (Bruker AXS, Karlsruhe, Germany). The X-ray diffractometer was operated at 40 kV and 40 mA to generate the Cu Kα radiation (1.5406 Å). Data were recorded from 5 to 70° in the 2θ range. Identification of the crystal phases was achieved by comparison of the XRD profile with the ICDD (International Centre for Diffraction Data, release 2018) database. The determination of the specific surface area (SSA) of the solids (m^2^/g) was carried out in the ChemiSorb 2720 equipment (Micromeritics, Norcross, GA, USA) coupled to temperature-programmed controller and software (TPx) by nitrogen adsorption at the temperature of liquid nitrogen (−196 °C) with a 30% gas mixture of N_2_ diluted in He. The Chemisoft TPx System (version 1.03; Data analysis software; Micromeritics, Norcross, GA, USA, 2011) allowed calculating the specific surface area using the Brunauer-Emmet-Teller (BET) equation and the single point method. Finally, the adsorbents were also characterized by the point of zero charges (pH_PZC_) at different pH values using a Jenway 7350 spectrophotometer (Cole-Parmer, Staffordshire, UK).

### 2.7. Adsorption Studies

The adsorption experiments were conducted using a batch method at room temperature. Typically, 25 mg of extruded adsorbents were magnetically stirred in a methylene blue aqueous solution (100 mL of water containing 20 mg mL^–1^ methylene blue). The remaining concentrations of methylene blue were determined at 623 nm using a Jenway 7350 spectrophotometer (Cole-Parmer, Staffordshire, UK). The adsorption rate of MB was calculated by the absorbance according to the Beer–Lambert law. Samples were drawn at 5 min intervals with a syringe and filtered through a 0.45 µm membrane filter to remove any solid particles interfering with the measurement. All tests were carried out in triplicate. The procedure was repeated using a methylene blue reference solution without extruded adsorbents to eliminate any photolysis effects causing discoloration of the solution due to natural light. The adsorbed quantity of methylene blue was calculated by Equation (1):

(1) $$q_{e}=\;\left(C_{0}-\;C_{e}\right)\times \frac{v}{w}$$

where *C*_0_ (mg L^−1^) and *C_e_* (mg L^−1^) represent the initial and equilibrium concentration, respectively; *v* (L) is the volume of solution and *w* (g) is the mass of adsorbent.

#### 2.7.1. Effect of pH

The effect of pH on MB adsorption onto the adsorbents was investigated under varying pH values from 3 to 10. The initial MB concentration used was 25 mg mL^–1^ for all extrudates. The contact time was fixed at 180 min and corresponded to the time necessary to reach adsorption equilibrium for all adsorbents. To evaluate the impact of pH on the solid surface, a point of zero charges pH_PZC_ measurement was also performed for all extrudates. The pH_PZC_ determinations were performed in aqueous suspensions of the extrudates at two concentrations (0.01 and 0.05 M) of the NaCl inert electrolyte. Potentiometric titrations were made over the entire pH range of 3–10.

#### 2.7.2. Isotherm Models

The effect of the initial MB concentration was investigated from 0.25 to 30 mg L^–1^. The experiments were performed without adjusting the pH of the solution. At the end of the experiments, the equilibrium pH was measured and found to be constant, around 7 for each adsorbent. The equilibrium sorption of MB was evaluated according to the Langmuir and Freundlich isotherm models, since these models can help to explain the adsorption mechanism and the heterogeneity of the adsorbent surface [15,48,57].

The expression of the Langmuir isotherm model can be expressed by Equation (2):

(2) $$\frac{C_{e}}{q}=\;\frac{1}{K_{L}q_{max}}+\;\frac{C_{e}}{q_{max}}$$

where *q_max_* is the maximum monolayer adsorption, *K_L_* is the equilibrium Langmuir constant related to the adsorption energy, and *C_e_* is the concentration of solute at equilibrium. Additionally, the *R_L_* separation factor values, which provide an insight into the nature of adsorption, can be expressed by Equation (3):

(3) $$R_{L}=\;\frac{1}{\left(1+K_{L}C_{e}\right)}$$

The expression of the Freundlich isotherm model can be represented by Equation (4):

(4) q= KFCe1n

where *K_F_* is the Freundlich constant, which indicates the adsorption affinity of the adsorbents and 1/*n* is another constant that represents the intensity of adsorption.

#### 2.7.3. Kinetic Models

The solute absorption rate of the solute–solution interface was described in this study using reaction-based models, called pseudo-first-order and pseudo-second-order, as well as diffusion-based models, called intraparticle diffusion, external-film diffusion, and internal-pore diffusion [2].

The pseudo-first- and second-order models assume that the difference between the average solid-phase concentration (*q_t_*) and the equilibrium concentration (*q_e_*) is the driving force for adsorption and that the overall adsorption rate is proportional to this driving force. Both equations have been widely applied to explain the experimental results obtained for aqueous pollutants such as dyes and metal ions [15,48,57]

The pseudo-first-order kinetic model is expressed by Equation (5):

(5) $$\ln\left(q_{e}-q_{t}\right)=\ln\left(q_{e}\right)-\;k_{1}t$$

where *k*_1_ is the rate constant (min^−1^), and *q_e_* and *q_t_* represent the MB adsorbed per unit weight (mg g^−1^) at equilibrium and at any time *t*, respectively.

The pseudo-second-order kinetic is expressed by Equation (6):

(6) $$\frac{t}{q_{t}}=\;\frac{1}{k_{2}q_{e}^{2}}+\;\frac{1}{q_{e}}t$$

where *k*_2_ is the pseudo-second-order rate constant (g mg^−1^ min^−1^).

The intraparticle diffusion model assumes that intraparticle diffusion is the rate control step, which is generally the case for well-mixed solutions [6]. The mathematical expression of the intraparticle diffusion model is described by Equation (7):

(7) qt= k3t12+A

where *k*_3_ (mg g^−1^ min^−1/2^) is the intraparticle diffusion rate constant and *A* (mg g^−1^) is a constant that indicates the thickness of the boundary layer, i.e., the higher the value of *A*, the greater the boundary layer effect. In some cases, the plot *q_t_* versus square root time can show multi-linearity, which indicates that several steps occur in the process.

The internal-pore diffusion model was also used to describe the kinetic sorption data. If particle diffusion controls (*D_p_*) the sorption rate is described using Equation (8):

(8) $$-ln\left(1-{\left(\frac{q_{t}}{q_{e}}\right)}^{2}\right)=\;\frac{2\pi^{2}D_{p}}{r^{2}}\;t$$

When the rate of sorption is controlled by external-film diffusion, it is expressed by Equation (9):

(9) $$-ln\left(1-\left(\frac{q_{t}}{q_{e}}\right)\right)=\;\frac{D_{f}C_{s}}{h\;r\;C_{z}}\;t$$

where *q_t_* and *q_e_* are the solute loadings on the adsorbent phase at time *t* and at equilibrium (mg g^−1^), respectively, *t* is the contact time (min), *C_s_* (mg L^−1^) and *C_z_* (mg kg^−1^) are the ion concentrations in the solution and in the adsorbent, respectively, *r* is the average radius of the adsorbent particles (1 × 10^−7^ m), and *h* is the film thickness around the adsorbent particles, accepted as 10^−6^ m for poorly stirred solutions [58]. *D_p_* is the diffusion coefficient in the adsorbent phase (m^2^ min^−1^) and D*_f_* (m^2^ min^−1^) is the diffusion in the film phase surrounding the adsorbent particles.

#### 2.7.4. Reuse of the Adsorbents

In this study, composites were desorbed after completing one treatment cycle and reused to determine their recycling property. MB desorption from composites loaded with this dye was verified using pure methanol and methanol solutions containing 6% (*v*/*v*) acetic acid as eluent. After desorption, the composites were dried and used in a new cycle under the same conditions as the previous cycle. The recycling experiments were carried out in three cycles.

## 3. Results

### 3.1. Characterization of the Compounds

#### 3.1.1. XRD Analysis

**Figure 1:**
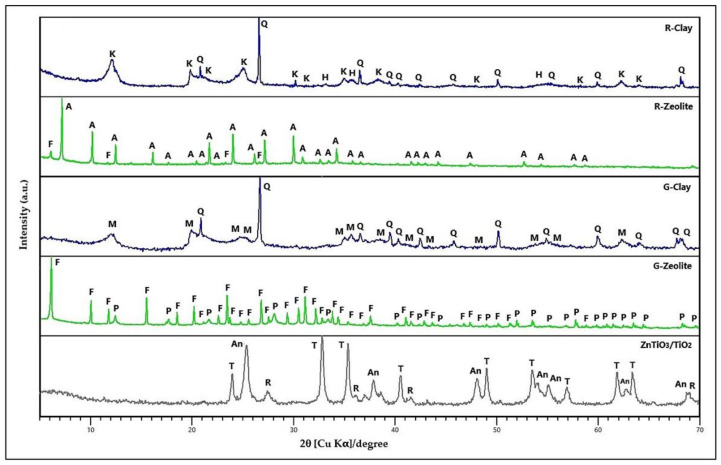
X-ray diffraction (XRD) pattern of R-Clay, R-Zeolite, G-Clay, G-Zeolite, and ZnTiO_3_/TiO_2_. K: Kaolinite, Q: Quartz, H: Hematite, M: Metahalloysite, Q: Quartz, F: FAU zeolite, A: LTA zeolite, P: Na-P1 zeolite, T: Titanate, An: Anatase, R: Rutile.

Figure 1 presents the XRD patterns of red clay (R-Clay), zeolites synthesized from this clay (R-Zeolite), gray clay (G-Clay), zeolites synthesized from this clay (G-Zeolite), and the ZnTiO_3_/TiO_2_ semiconductor. These compounds were used as precursors to prepare extruded composites.

R-Clay consists of quartz (Q), kaolinite (K) and hematite (H). R-Zeolite consists of LTA, and FAU zeolites. Figure 1 shows the main diffraction peaks of LTA zeolite of the sodium form (Table S1). The LTA zeolite was indexed to cubic phase with unit cell parameters a = b = c = 24.61 Å and space group Fm3c(226) according to the standard ICDD card No: 39-0222. Figure 1 also shows the main diffraction peaks of the FAU zeolite of the sodium form (Table S1). The FAU zeolite was indexed to cubic phase with unit cell parameters a = b = c = 25.028 Å and space group Fd-3(203) according to the standard ICDD card No: 39-0218. The zeolites presented a percentage of crystallinity of 67% compared to the standards. On the other hand, G-Clay consists of quartz (Q) and metahalloysite (M). G-Zeolite consists of FAU and Na-P1 zeolites. Figure 1 shows the main diffraction peaks of the FAU zeolite of the sodium form (Table S1). The FAU zeolite was indexed to cubic phase with unit cell parameters a = b = c = 25.028 Å and space group Fd-3(203) according to the standard ICDD card No: 39-0218. The number of structural Al atoms in the FAU and the structural Si/Al ratio of this zeolite were calculated using the Breck–Flanigen equation [59]. The results obtained (N_Al_ = 89 and Si/Al = 1.2) classify the synthesized FAU zeolite as type X [27]. Figure 1 also shows the main diffraction peaks of the Na-P1 zeolite (Table S1). The Na-P1 zeolite was indexed to tetragonal phase with unit cell parameters a = b = 9.999 Å and c = 10.069 Å and space group I4_1_/amd(141) according to the standard ICDD card No: 44-0052. The zeolites presented a percentage of crystallinity of 63% compared to the standards. Finally, Figure 1 shows the main diffraction peaks of the ZnTiO_3_/TiO_2_ heterostructure (Table S1). The ZnTiO_3_/TiO_2_ heterostructure nanomaterial obtained was indexed to hexagonal phase with unit cell parameters a = b = 5.08 Å and c =13.93 Å and space group R-3(148) according to the standard JCPDS card No. 00-015-0591 for the ZnTiO_3_ phase. The TiO_2−a_ species was indexed to tetragonal phase with unit cell parameters a = b = 3.79 Å and c = 9.51 Å and space group I4_1_/amd(141) according to the standard JCPDS card No. 01-073-1764. The TiO_2−r_ phase was assigned to tetragonal phase with unit cell parameters a = b = 5.08 Å and c = 13.93 Å and space group P42/mnm(136) according to the standard JCPDS card No. 03-065-0192.

When comparing the diffraction patterns of the extruded composites with their respective constituent compounds (Figures S1 and S2), no alteration of the diffraction peaks of the zeolites or the mixed oxide ZnTiO_3_/TiO_2_ was observed, probably due to the calcination temperature of the extruded composites being lower than the thermal stability of the zeolites [60] and mixed oxide [26] used. Therefore, the calcination temperature of 500 °C made it possible to achieve a mechanically strong adsorbent while keeping the crystalline structure of the zeolites and the photocatalyst intact.

#### 3.1.2. SEM-EDX Analysis

**Figure 2:**
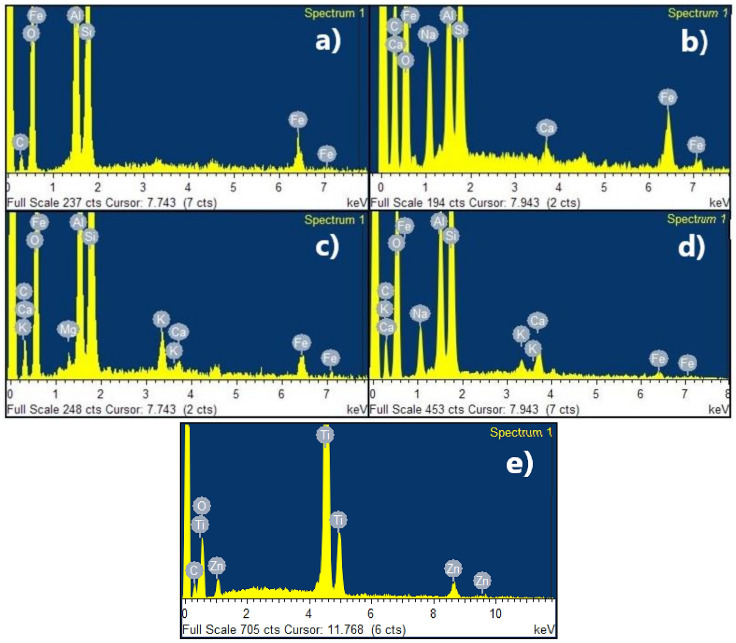
Energy dispersive X-ray (EDX) spectra of (**a**) R-Clay, (**b**) R-Zeolite, (**c**) G-Clay, (**d**) G-Zeolite and (**e**) ZnTiO_3_/TiO_2_.

Figure 2 presents the EDX (energy dispersive X-ray) spectra of clays and zeolites obtained from these clays, indicating the presence of several elements such as C, Al, Si, Fe, Ca, Na, K, Mg, and O. G-Clay and G-Zeolite had more exchange cations than R-Clay and R-Zeolite; however, R-Clay and R-Zeolite had a larger amount of Fe than G-Clay and G-Zeolite. In R-Clay the Fe was in the form of hematite, according to the diffraction pattern shown in Figure 1. Zeolites had a higher amount of sodium than their respective clays due to the calcination treatment carried out before synthesis. Figure 2 also shows the EDX spectra of the ZnTiO_3_/TiO_2_ heterostructure, indicating the presence of O, Zn and Ti.

Table 1 shows the elemental composition (%) analyzed by EDX for the clays and zeolites obtained from these clays and ZnTiO_3_/TiO_2_.

**Figure 3:**
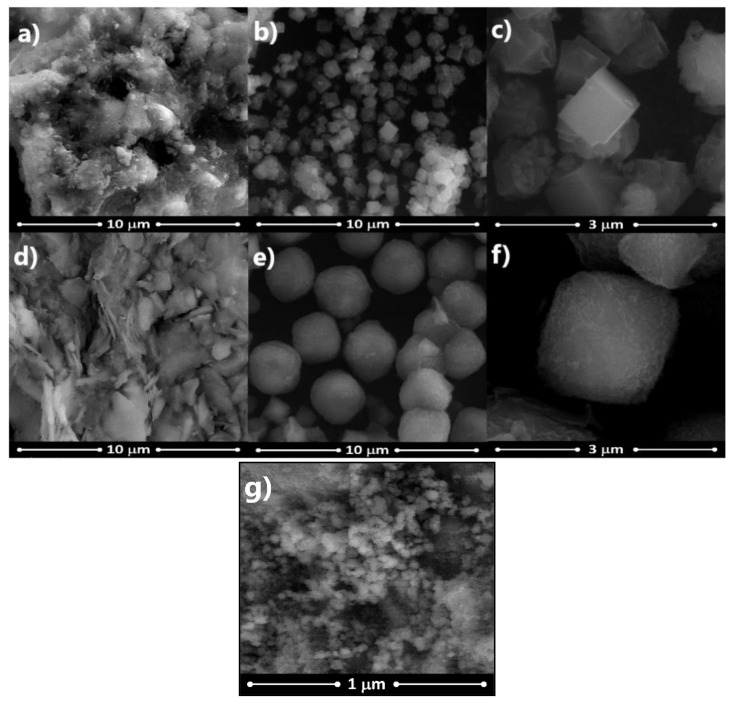
Scanning electron microscopy (SEM) images of (**a**) R-Clay, (**b**) and (**c**) R-Zeolite, (**d**) G-Clay, (**e**) and (**f**) G-Zeolite, (**g**) ZnTiO_3_/TiO_2_.

Figure 3 shows the SEM photomicrograph of clays and zeolites obtained from these clays. Figure 3a shows intertwined hematite crystals with a “cauliflower” habit coating kaolinite of the R-Clay, while in Figure 3b,c uniformly distributed granules are observed with a perfect morphology, typical of the LTA phase, and non-uniform agglomerates of nanocrystals are also observed on the well-formed LTA zeolite crystals, which can be attributed to the growth of the FAU phase during the synthesis of the zeolite. Additionally, the SEM photomicrograph in Figure 3d shows aggregates of G-Clay with varied morphology and sizes and with a very rough-appearing surface due to the conformation similar to “stacked fibers” that are a consequence of the possible grouping of halloysite nanotubes. In Figure 3e,f, the presence of uniformly distributed cubic spheroidal granules is observed, which correspond to the FAU zeolite. The particle sizes determined by SEM for both LTA and FAU zeolites were 1.6 and 3.9 µm, respectively. The sizes were determined using ImageJ, which is a powerful, oft-referenced program for image processing [61]. Finally, Figure 3g shows the SEM image of the ZnTiO_3_/TiO_2_ heterostructure. The image shows that the particles have a particle size less than 100 nm, are almost spherical and are highly agglomerated.

#### 3.1.3. XRF Analysis

Table 2 shows the main oxides present in the clays, zeolites, and compounds investigated in this study. In Table 2, the XRF analysis showed the majority presence of the cations Al, Si, Fe, Ca, K, Mg, and O in all the compounds, in addition to Ti and Zn, which were mainly incorporated as a mixed oxide of ZnTiO_3_/TiO_2_ in extrudates.

#### 3.1.4. Specific Surface Area (SSA) Analysis

The specific surface area of the adsorbents in both powder and extrudate form are summarized in Table 3. The prepared extruded composites had a smaller surface area compared to that of adsorbents in powder form due to the heat treatment required for their preparation. Despite the reduction in the specific surface area in the extrudates, the presence of exchange cations in their structure could contribute to the elimination of the dye from the solution since different mechanisms participate in the adsorption process.

### 3.2. Adsorption of MB

**Figure 4:**
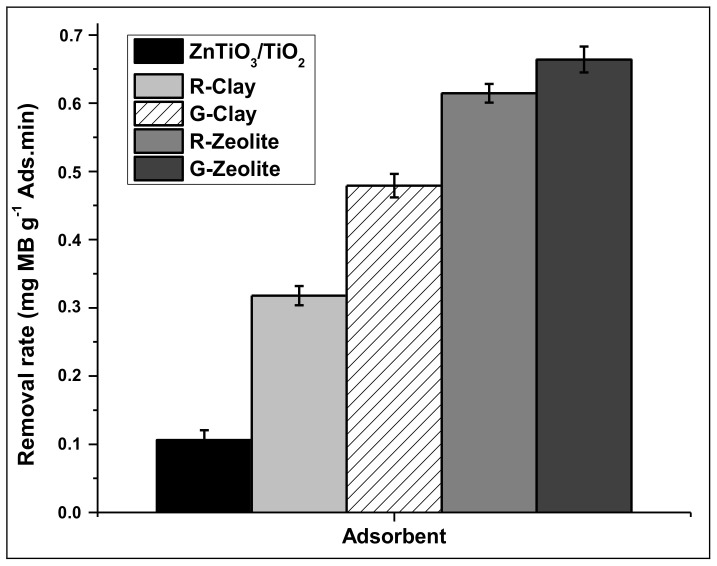
Methylene blue (MB) adsorption capacity of the ZnTiO_3_/TiO_2_ semiconductor, R-Clay, R-Zeolite, G-Clay, and G-Zeolite.

Figure 4 shows that G-Zeolite and G-Clay had a higher adsorption capacity of the MB dye than R-Zeolite, R-Clay, and the ZnTiO_3_/TiO_2_ semiconductor. The results corresponded to the evaluation of powder samples. As expected, zeolites had better adsorption capacity than clays and mixed oxide of Zn and Ti. Additionally, G-Zeolite had better MB adsorption capacity than R-Zeolite and, similarly, G-Clay had better MB adsorption capacity than R-Clay. In addition, the higher adsorption capacity of G-Zeolite compared to R-Zeolite may be due to the much larger pore of FAU zeolite compared to LTA zeolite.

#### 3.2.1. Effect of pH

**Figure 5:**
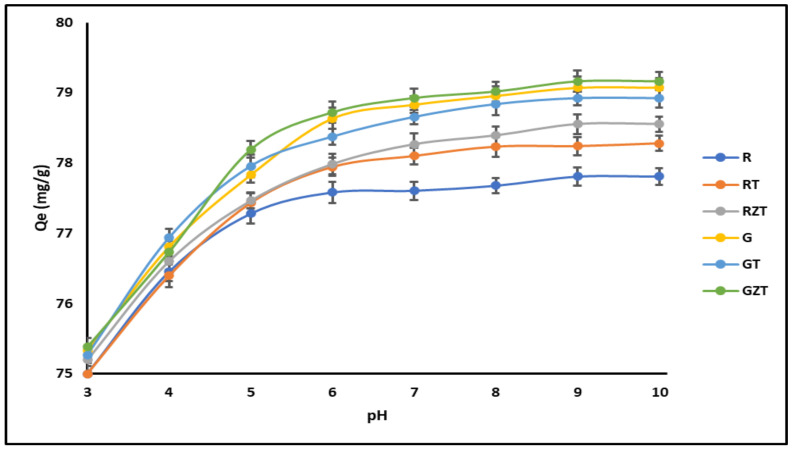
Effect of pH on the adsorption of MB onto composites.

The R and G extrudates showed pH_PZC_ values around 4.0, while the GT, GZT, RT, and RZT extrudates showed pH_PZC_ values around 6.0. At a pH higher than pH_PZC_, the surface had a net negative charge and the adsorption of the cationic dye molecule was promoted. However, MB adsorption was reduced at a pH lower than pH_PZC_ due to the net positive charge on the surface, which causes electrostatic repulsion. Figure 5 shows this effect of pH on (G) G-Clay, (GT) G-Clay-ZnTiO_3_/TiO_2_, (GZT) G-Clay-Zeolite-ZnTiO_3_/TiO_2_, (R) R-Clay, (RZ) R-Clay-ZnTiO_3_/TiO_2_ and (RZT) R-Clay-Zeolite-ZnTiO_3_/TiO_2_.

From the minimal increment in adsorption of MB in the solution at pH values above 8, it was decided that adsorption at pH 7 was the optimum operating condition for adsorption experiments.

#### 3.2.2. Adsorption Isotherm

**Figure 6:**
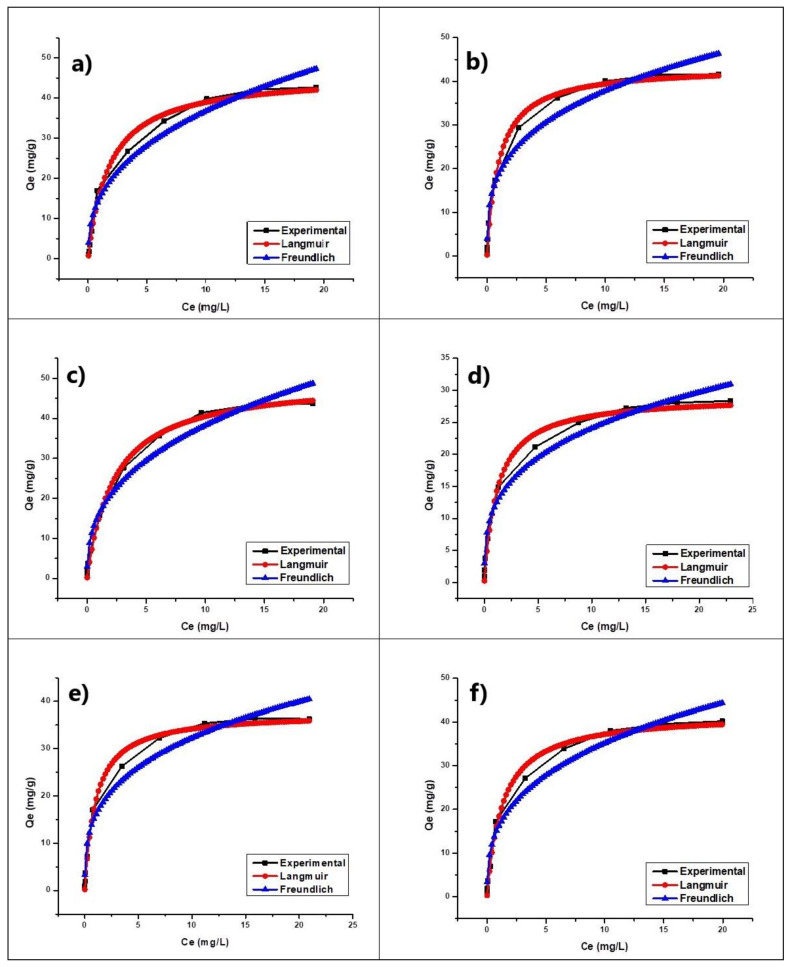
Absorption isotherm of (**a**) G, (**b**) GT, (**c**) GZT, (**d**) R, (**e**) RT and (**f**) RZT.

Figure 6 shows the adsorption isotherms of the extruded composites: (G) G-Clay, (GT) G-Clay-ZnTiO_3_/TiO_2_, (GZT) G-Clay-Zeolite-ZnTiO_3_/TiO_2_, (R) R-Clay, (RZ) R-Clay-ZnTiO_3_/TiO_2,_ and (RZT) R-Clay-Zeolite-ZnTiO_3_/TiO_2_. In this figure, it is evident that the Langmuir model is better than the Freundlich model to describe the behavior of all composites.

Table 4 shows the equilibrium data of MB sorption by extruded composites (G) G-Clay, (GT) G-Clay-ZnTiO_3_/TiO_2_, (GZT) G-Clay-Zeolite-ZnTiO_3_/TiO_2_, (R) R-Clay, (RZ) R-Clay-ZnTiO_3_/TiO_2_ and (RZT) R-Clay-Zeolite-ZnTiO_3_/TiO_2_. Furthermore, the *R_L_* separation factor or equilibrium parameter was calculated using Equation (3), obtaining low *R_L_* values for all the adsorbents. When 0 < *R_L_* < 1, favorable adsorption was indicated, and *R_L_* > 1 meant unfavorable adsorption; *R_L_* = 0 indicated irreversible adsorption, and *R_L_* = 1 meant energy dispersive X-ray linear adsorption [11].

#### 3.2.3. Adsorption Kinetics

**Figure 7:**
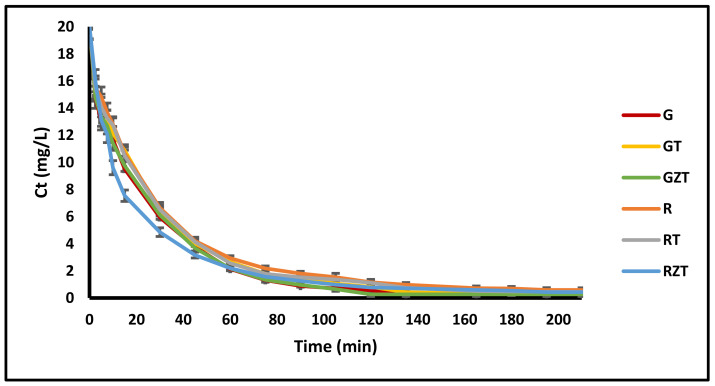
Time-course variation of the composites’ *C_t_* (mg L^–1^) curves.

Figure 7 shows the time-course variation of concentration *C*_t_ (mg L^–1^) curves of the extruded composites (G) G-Clay, (GT) G-Clay-ZnTiO_3_/TiO_2_, (GZT) G-Clay-Zeolite-ZnTiO_3_/TiO_2_, (R) R-Clay, (RZ) R-Clay-ZnTiO_3_/TiO_2_ and (RZT) R-Clay-Zeolite-ZnTiO_3_/TiO_2_. The figure shows that the MB concentration in the solution decreased rapidly around the first 60 min, after which removal tended to become constant.

**Figure 8:**
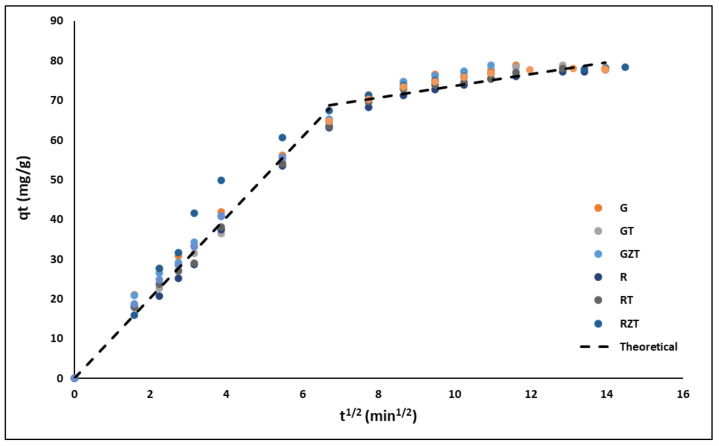
Intra-particle diffusion plots for MB removal by the extrudates.

The intra-particle diffusion model fitted well the experimental data, as can be seen in Figure 8, indicating that the entire sorption process was divided into two linear regions. Hence, the MB sorption process might be described by film diffusion followed by a particle diffusion process [6].

Table 5 shows the equilibrium data of MB sorption by the extruded composites (G) G-Clay, (GT) G-Clay-ZnTiO_3_/TiO_2_, (GZT) G-Clay-Zeolite-ZnTiO_3_/TiO_2_, (R) R-Clay, (RZ) R-Clay-ZnTiO_3_/TiO_2_ and (RZT) R-Clay-Zeolite-ZnTiO_3_/TiO_2_.

### 3.3. Reuse of the Adsorbents

**Figure 9:**
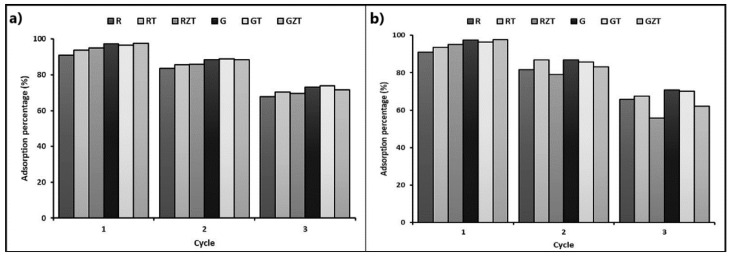
Adsorption percentage of MB for three successive adsorption cycles. (**a**) Composites desorbed with methanol; (**b**) composites desorbed with an acid solution.

Figure 9 shows the MB adsorption capacity for three cycles of the extruded composites (G) G-Clay, (GT) G-Clay-ZnTiO_3_/TiO_2_, (GZT) G-Clay-Zeolite-ZnTiO_3_/TiO_2_, (R) R-Clay, (RZ) R-Clay-ZnTiO_3_/TiO_2,_ and (RZT) R-Clay-Zeolite-ZnTiO_3_/TiO_2_. The figure shows that the adsorption capacity of composites containing zeolite decreased more than in extrudates without zeolite after the first and second regeneration cycles when an acid solution was used for desorption.

## 4. Discussion

### 4.1. Synthesis and Characterization of Compounds

Firstly, the structural identification of the clays, zeolites, and mixed oxide of Zn and Ti was performed using XRD. When comparing in Figure 1 the diffraction patterns of the zeolites with the respective clays, disappearance of the quartz reflections and the other mineralogical phases present in the clays was observed. Next, the diffraction patterns of the zeolites evidenced the appearance of peaks corresponding to the LTA-FAU and FAU-NaP_1_ phases. The presence of these phases revealed the transformation of the clays, which could begin with the formation of an amorphous material followed by the subsequent co-crystallization of the LTA-FAU and FAU-NaP_1_ zeolitic phases. On the one hand, these results are consistent with the metastable character of the zeolitic phases obtained; in agreement with the literature, the mix of stable metaphases was favored by the differences in the Si/Al ratio and the effect of the cations present in the original clays [62,63,64]. On the other hand, the percentages of the crystalline phase of R-Zeolite and G-Zeolite were determined using XRD, being 67% and 63%, respectively. In general, the amorphous phase formation can be related to the presence of geopolymers [65,66]. Specifically, geopolymers can be identified as the amorphous equivalent of crystalline structures of aluminosilicates with an equal chemical composition of the zeolites, but those presented a disordered structure unlike the ordered structure of zeolites [67,68,69]. Regarding the results, the diffraction pattern showed that R-Clay was formed from kaolinite (K), quartz (Q), and hematite (H), while G-Clay consisted mainly of metahalloysite (M) and quartz (Q). Furthermore, Figure 1 shows the diffraction pattern of the ZnTiO_3_/TiO_2_ heterostructure, which was obtained without impurities, as we reported in previous works [26].

In this study, clays and zeolites were also characterized by EDX to determine their chemical composition. In previous works, the characterization by EDX of the mixed oxide of Zn and Ti was presented. From the EDX results shown in Figure 2, the change in the weight percentage of the synthesized materials was observed after the treatment carried out to obtain zeolites. In general, the impurities of the starting clays decreased either by the action of the thermal and hydrothermal treatments and/or by dilution effects after the addition of new components in the synthesis gel, such as alumina and NaOH [70]. However, the percentages of Na^+^ increased significantly due to the incorporation of NaOH as a mineralizing agent in the synthesis of the zeolites [71,72]. This specific fact is due to the capture of Na^+^ ions by the zeolitic structure, to neutralize the negative charge of aluminum in the zeolite and/or geopolymer when they have been formed [73]. On the other hand, from the XRF analysis, the presence of various oxides in the sorbents studied was determined. Table 2 shows that the sorbents were mainly made up of SiO_2_, Al_2_O_3_, TiO_2_, ZnO, as well as lower percentages of Fe_2_O_3_, K_2_O, CaO, and MgO. These oxides provided qualities to the sorbents to improve the adsorption of methylene blue by cation exchange.

The morphological and textural characterization of the clays and zeolites was performed to have a more complete characterization map of these solids. The morphology of the clays and zeolites was identified by scanning electron microscopy (SEM). The SEM photomicrographs obtained and presented in Figure 3 show the morphology of the clays according to their mineralogical composition. Likewise, Figure 3 shows the typical morphology of the LTA and FAU phases, which are similar to those described by various authors for these phases [74,75,76].

To characterize the textural properties of the materials, the specific surface area of the synthesized materials was determined by the physisorption of N_2_. The results listed in Table 2 show that the extrudate materials had a lower specific surface area than the powdered ones. Probably, the thermal conditions used in the calcination of the extrudates reduced their surface area [77,78].

The decrease in the surface area of the extruded adsorbents is essentially attributed to the elimination of some of the constituents from the internal surface of the clay (e.g., adsorbed species) after the calcination process. The removal of these constituents created additional spaces within the wide pores of the clay structure and also resulted in a reduction of the internal surface area probably due to shrinkage. It is believed that this behavior, due to the increase in temperature, occurred due to the elimination of the physisorbed water as well as the superficial hydroxyl groups weakly attached to the clay structure [79]. In the present study, the addition of zeolites as a porous material to the extruded adsorbents did not improve their MB adsorption capacity from the aqueous solution. Although clay used as an inorganic binder provided strength and wear resistance in extruded adsorbents, the incorporation of clay can dilute the active porous component (i.e., the zeolite), resulting in a reduced specific surface area. Also, the clay could coat the surface of the zeolite and cause the pores to become blocked. Consequently, the MB removal capacity in aqueous systems of extrudates having zeolite did not improve compared to extrudates free of zeolite. These results are in argument with that reported by Akhtar et al. [39]

Although usually the powdered materials have a higher specific surface area, in this study the extrudates were chosen to adsorb MB due to their appropriate mechanical and chemical stability, which facilitated their recovery at the end of the process and their reuse after several cycles.

### 4.2. Adsorption of MB

Preliminary batch adsorption of MB was performed from an aqueous solution to investigate the adsorption properties of clays, zeolites, and ZnTiO_3_/TiO_2_, which were used in powder form. Figure 4 shows that zeolites had the highest adsorption capacity. This great adsorption capacity was because zeolites have a porous structure and therefore a higher specific surface than clays, so they can easily host large molecules on their surface [80,81]. On the other hand, the extrudates showed a lower specific surface area, but were also effective in removing MB from the aqueous solution, probably through other mechanisms, including electrostatic interaction, chemical reactions such as complexation, or ion exchange between sorbent and MB [82]. Consequently, despite the reduction in specific surface area in the extrudates, the surface chemistry of these materials was also an important factor controlling MB adsorption. The extruded clays showed a pH_PZC_ value around 4, and the adsorption tests were carried out at pH = 7.0, so the surface of these materials was negatively charged, improving the adsorption of cationic dye. Also, according to Table 1 and Table 2, clays and zeolites contained various cations, such as Mg, K, Ca, Na, and Fe, that could promote the cation exchange capacity of prepared extruded composites to improve their MB adsorption capacity [83]. In addition to that, G-Clay contains metahalloysite (M), a mineralogical phase with applications in the design and preparation of materials with adsorbent properties [84].

Since the absorption process occurs by different mechanisms, several batch adsorption experiments of MB from an aqueous solution were developed to investigate the performance of the G, GT, GT, R, RT, and RZT extrudates. The experiments were developed varying the following parameters: the initial pH of the MB solution, the initial MB concentration, and the contact time.

#### 4.2.1. Effect of pH

A factor affecting the adsorption of dyes is the pH of the solution. pH affects the adsorbent surface charge, the electrical charge of the dye, and the degree of ionization, which control the adsorption process. It is expected that adsorption will increase with pH, particularly for an adsorbate with a cationic nature [71]. At pH values above pH_PZC_, the surface has a net negative charge and tends to accumulate cationic dye molecules due to the electrostatic attraction between the cationic dye molecule and the negatively charged extrudate surface, as suggested by other authors [85]. However, MB adsorption is reduced at a pH lower than pH_PZC_ due to the net positive charge on the surface, which causes electrostatic repulsion. As shown in Figure 5, the amount of MB adsorbed increased when the pH increased from 3.0 to 9.0. However, an increase in adsorption at pH values between 7.0 and 9.0 was relatively lower than the increase in adsorption at pH values between 3.0 and 7.0. The high adsorption capacity observed at alkaline pH values was due to the increase in hydroxyl ions and, therefore, to the increased electrostatic attraction between the positive and negative charges of the adsorption sites [11]. However, at very alkaline pH levels, it appears that OH ions formed a complex with other ions within alkaline pH ranges, affecting the adsorption of the dye by the adsorbent [3]. This could lead to precipitation of MB on the adsorbent surface, since the adsorption process was probably a combination of electrostatic attraction, sorption, and precipitation [82].

#### 4.2.2. Adsorption Isotherm

The results of the adsorption isotherm studies showed that using the extrudates, the MB removal rate first increased from 0.25 to 20 mg L^–1^, and that it then was reduced when the initial MB concentration (20–30 mg L^−1^) was increased. This can be explained by the fact that, at higher concentrations, more MB molecules were competing for the active sites available on the surface of the adsorbent material. These active sites, which were in limited quantity, were quickly saturated when the concentration of MB increased. Therefore, the initial concentration of dye provided a significant driving force to overcome the mass transfer resistance of the dye between the aqueous solution and the surface of the extrudates [2].

The parameters corresponding to the fitting of these results to the Langmuir and Freundlich isotherm models are summarized in Table 3. The experimental data of the adsorption seemed fit to Langmuir and Freundlich isotherm models. The correlation coefficients in both isotherm models were near 1, indicating that the two models fit the experimental data well [86]. However, as shown in Figure 6, Langmuir isotherm models gave better fitting than Freundlich isotherm models. It can be concluded that adsorption of MB onto these adsorbents is considered as monolayer adsorption rather than as multi-layer adsorption. This fact supposes that the adsorption of MB on the extrudates occurs as a phenomenon of electrostatic attraction where the adsorption energy is uniform [87]. During this adsorption process, the cationic dye tends to move through the pores and channels of the extrudates, replacing the exchangeable cations present in the synthesized materials, which are shown in Table 1.

#### 4.2.3. Adsorption Kinetics

Although the adsorption models help to establish the efficiency in the process, it is also important to determine the kinetic mechanism. The adsorption kinetic models express the contact time required for the complete adsorption of the chemical species. From them, we can establish the optimal conditions for a process of continuous dye removal and/or scaling at an industrial level. Figure 7 illustrates the concentration of MB in an aqueous solution at different contact times. It was observed that MB concentration decreased rapidly at the initial adsorption process, and then was followed by a slow reduction beyond 60 min for all adsorbents. From this trend, we can conclude that the equilibrium was reached at the contact time of around 180 min. The rapid initial stage of adsorption resulted from the presence of the vacant adsorption sites as well as the presence of a high concentration gradient. On the one hand, the adsorption by all extrudates can be attributed to the negative surface charge of these materials, leading to a high electrostatic attraction between the negatively charged sorbents and the positively charged cationic MB [88]. On the other hand, the efficiency of extrudates GT, GZT, RT, and RZT to adsorb dissolved MB dye molecules is also attributed to the presence of mixed oxide ZnTiO_3_/TiO_2_ nanoparticles, which provided additional active sites for the chemical adsorption of the dye.

The adsorption kinetic parameters are summarized in Table 4. The highest correlation coefficient (R^2^) was obtained for the pseudo-second-order model; this kinetic model was the one that best fits in this study. The mechanism described by the pseudo-second-order model indicated a chemical adsorption of the cationic dye in the extrudates [89]. On the other hand, in Figure 8 two linear regions were identified when the experimental data were fitted to the intraparticle diffusion model, suggesting that the MB sorption process could be described by external-film diffusion followed by internal-pore diffusion. Table 4 also summarizes the linear regression analysis for the diffusion kinetic models. The highest values of the regression coefficient (R^2^) were found for the external-film diffusion; furthermore, the values of A were relatively high, therefore, the surface adsorption was the rate-limiting step [5].

### 4.3. Reuse of the Adsorbents

The mechanical stability of structured materials is directly related to their useful life. If the mechanical stability is poor, the material will gradually disintegrate in the reaction solution, causing loss of activity and contamination of the medium. Mechanical stability correlates with calcination temperature [90]. The higher the calcination temperature, the better the mechanical stability, but there is an optimal calcination temperature for maximum mechanical stability. In this work, a maximum calcination temperature of the extrudates of 500 °C was used to avoid the change of crystalline phase of the synthesized compounds. The extruded materials thus prepared were subjected to desorption and regeneration studies to determine their economic viability in the MB adsorption process. The maximum desorption of cationic dye was obtained in acid solution, which indicates that the adsorption was performed through an ion exchange process. Regeneration studies were carried out for three regeneration cycles. However, as shown in Figure 9, the adsorption capacity of extrudates containing zeolite decreased more than in extrudates without zeolite after the first and second regeneration cycles when an acidic solution was used for desorption. This reduction in adsorption capacity could be due to decomposition or damage caused by the acid solution to certain adsorption sites or functional groups present on the surface of the extruded composites.

### 4.4. MB Adsorption Capacity of the Synthesized Compounds Compared to Other Compounds Described in the Literature

Table 6 shows that the compounds synthesized in this article were effective in removing dyes in aqueous effluents, and the reasons were probably due to the combined effects of several factors.

Table 6 shows that the presence of the mixed oxide ZnTiO_3_/TiO_2_ did not significantly improve the MB adsorption capacity of the extrudates compared to the extrudates free of ZnTiO_3_/TiO_2_. However, this mixed oxide can act as a photocatalyst to improve MB removal in aqueous systems when the process is carried out under UV or solar light, as we reported in a previous study [26]. The efficiency of ZnTiO_3_/TiO_2_ in the photocatalytic removal of MB in aqueous systems depends on the appropriate contact between the photocatalyst and the dye. Consequently, the extruded adsorbents reported in this study not only acted as supports for the photocatalyst, but also efficiently captured the contaminant on its surface to initiate the oxidative process. Therefore, the addition of ZnTiO_3_/TiO_2_ in the extruded adsorbents will allow in the future combining both processes, adsorption and photocatalysis, to improve the removal of MB from aqueous systems.

Similarly, Table 6 shows that the zeolites did not improve the MB removal capacity of the extruded composites, despite the fact that the zeolites in powder form showed greater dye removal capacity than the precursor clays and the mixed oxide ZnTiO_3_/TiO_2_. This is because extruded zeolites have a lower specific surface area than powdered zeolites, significantly affecting their ability to remove dye. In the present study, the zeolites were obtained from two Ecuadorian clays. These clays by themselves were able to remove MB through different mechanisms, so from an economic and environmental point of view, clays are ideal for wastewater treatment. Clays were also used as inorganic binders to prepare structured materials with adequate mechanical strength and wear resistance. Consequently, clays proved to be materials that can offer universal and commercial opportunities to prepare sustainable materials with improved properties for various applications.

## 5. Conclusions

From hydrothermal synthesis and alkaline fusion methods, zeolites were successfully synthesized from Ecuadorian clays, obtaining combinations of zeolites: LTA-FAU and FAU-NaP1, which showed good textural and morphological characteristics suitable for adsorption processes.

The zeolites were combined with their precursor clays and the mixed oxide of Zn and Ti to prepare extrudates that were successfully used as adsorbents in methylene blue removal tests, determining the adsorption capacity and the kinetic model for the removal of MB of each synthesized material. In general, the experimental isotherms were fitted to the Langmuir model, which describes a monolayer adsorption on a surface containing an infinite number of identical sites. This model has correlated with the pseudo-second-order kinetic model found, which indicates a chemisorption process on the adsorbent. It is clear then that the adsorption of the cationic dye increases rapidly until reaching a surface saturation in the structure due to two phenomena in particular. In the first of them, it is suggested that there is a comparable concentration of cationic species in the active centers of the extrudates that can be exchanged with the dye in question. On the other hand, the presence of MB complexes increases the adsorption of the cationic dye on the surface of the extrudates.

In particular, it was evidenced that extrudates derived from gray clay were the materials that showed the highest performance in the MB removal process. This fact was evidenced by the high adsorption capacity (*q_m_*) determined in the Langmuir model. In the same way, as shown by the adsorption rate constant in the pseudo-second-order model, the removal percentages of the MB decreased due to the surface saturation of the extrudates.

In this way, the usefulness of a natural resource such as clays and their transformation into higher value-added products such as zeolites was corroborated. The adsorption capacity and efficiency of these materials combined with ZnTiO_3_/TiO_2_ was demonstrated in the process of MB removal from aqueous solutions, which leaves a door open to the potential generation of clean technologies at an industrial scale from available natural resources.

## Figures and Tables

**Table 1:** Elemental analysis (wt%) for clays and zeolites.

	C	O	Si	Al	Fe	Na	Ca	K	Mg	Ti	Zn	O
R-Clay	12.10	47.71	19.08	15.40	5.72	-	-	-	-	-	-	-
R-Zeolite	32.43	47.89	6.49	5.01	3.91	3.79	0.48	-	-	-	-	-
G-Clay	16.99	46.46	18.17	12.22	3.27	-	0.54	1.84	0.53	-	-	-
G-Zeolite	20.77	50.91	14.44	6.77	1.88	2.70	1.88	0.65	-	-	-	-
ZnTiO_3_/TiO_2_	5.42	-	-	-	-	-	-	-	-	54.85	6.13	33.60

**Table 2:** Composition (%) of clays, zeolites, and composites.

Compound	Al_2_O_3_	SiO_2_	K_2_O	CaO	TiO_2_	MgO	Fe_2_O_3_	ZnO
G-Clay	21.20 (±0.85)	45.60 (±0.68)	1.63 (±0.03)	0.22 (±0.01)	0.39 (±0.01)	0.16 (±0.00)	1.94 (±0.01)	0.01 (±0.00)
G-Zeolite	19.60 (±0.79)	41.30 (±0.63)	0.49 (±0.02)	4.11 (±0.02)	0.19 (±0.01)	0.07 (±0.00)	0.70 (±0.01)	0.06 (±0.00)
GT	11.60 (±1.56)	12.20 (±0.53)	0.32 (±0.03)	1.59 (±0.02)	51.30 (±0.13)	0.04 (±0.01)	3.64 (±0.03)	18.95 (±0.05)
GZT	18.00 (±1.20)	34.60 (±0.68)	1.58 (±0.04)	2.67 (±0.03)	28.10 (±0.08)	0.07 (±0.01)	2.38 (±0.02)	12.29 (±0.03)
R-Clay	27.10 (±0.98)	39.40 (±0.65)	0.78 (±0.02)	0.12 (±0.01)	1.01 (±0.01)	0.09 (±0.00)	7.81 (±0.02)	0.01 (±0.00)
R-Zeolite	18.60 (±0.99)	27.60 (±0.59)	-	1.52 (±0.02)	1.09 (±0.01)	0.08 (±0.00)	6.72 (±0.02)	0.08 (±0.00)
RT	8.88 (±1.62)	13.90 (±0.58)	1.19 (±0.04)	1.69 (±0.02)	51.40 (±0.14)	-	2.09 (±0.03)	20.66 (±0.06)
RZT	10.10 (±1.31)	15.60 (±0.58)	0.38 (±0.59)	2.20 (±0.50)	31.30 (±1.82)	0.06 (±0.16)	7.87 (±0.69)	16.30 (±0.07)

**Table 3:** Specific surface area (SSA) (m^2^/g) of clays, zeolites, and composites.

Adsorbent	Composition	Form	SSA (m^2^/g)
R-Clay	Red clay	Powder	48.8
R-Clay (R)	Red clay	Extrudate	29.3
R-Zeolite	LTA/FAU zeolites	Powder	104
R-Composite	Red clay + ZnTiO_3_/TiO_2_	Powder	82.5
R-Composite (RT)	Red clay + ZnTiO_3_/TiO_2_	Extrudate	29.5
R-Composite	Red clay + LTA/FAU zeolites + ZnTiO_3_/TiO_2_	Powder	84.1
R-Composite (RZT)	Red clay + LTA/FAU zeolites + ZnTiO_3_/TiO_2_	Extrudate	31.1
G-Clay	Gray clay	Powder	42.8
G-Clay (G)	Gray clay	Extrudate	25.5
G-Zeolite	FAU/Na-P1 zeolites	Powder	349
G-Composite	Gray clay + ZnTiO_3_/TiO_2_	Powder	184
G-Composite (GT)	Gray clay + ZnTiO_3_/TiO_2_	Extrudate	25.8
G-Composite	Gray clay + FAU/Na-P1 zeolites + ZnTiO_3_/TiO_2_	Powder	188
G-Composite (GZT)	Gray clay + FAU/Na-P1 zeolites + ZnTiO_3_/TiO_2_	Extrudate	26.4

**Table 4:** Isotherm parameters for MB sorption on composites.

Isotherm Parameters		R	RT	RZT	G	GT	GZT
Langmuir	*q_max_* (mg g^−1^)	29.14 (±0.97)	37.59 (±0.98)	42.00 (±1.16)	45.88 (±1.65)	43.40 (±1.33)	49.81 (±1.33)
	*K_L_* (L mg^−1^)	0.82 (±0.15)	1.00 (±0.14)	0.77 (±0.11)	0.56 (±0.09)	0.99 (±0.17)	0.43 (±0.05)
	*R_L_*	0.06	0.05	0.06	0.08	0.05	0.10
	*χ* ^2^	2.00	2.11	2.34	3.36	3.38	1.60
	R^2^	0.98	0.99	0.99	0.99	0.99	0.99
Freundlich	*K_F_* (L mg^−1^)	11.98 (±0.92)	15.88 (±1.46)	16.16 (±1.55)	15.26 (±1.62)	18.80 (±1.58)	16.12 (±1.59)
	*n*	3.30 (±0.32)	3.25 (±0.39)	2.96 (±0.34)	2.62 (±0.30)	3.30 (±0.37)	2.66 (±0.29)
	1/*n*	0.30	0.31	0.34	0.38	0.30	0.38
	χ^2^	3.81	9.84	11.10	12.40	11.50	11.10
	R^2^	0.97	0.96	0.96	0.96	0.96	0.97

**Table 5:** Kinetic parameters for MB removal on composites.

Kinetic Parameters		R	RT	RZT	G	GT	GZT
Pseudo-first-order	*q_max_* (mg g^−1^)	75.47 (±1.15)	75.84 (±1.35)	75.50 (±1.04)	76.43 (±1.73)	76.57 (±1.42)	76.37 (±1.87)
	*k*_1_ (L mg^−1^)	0.05 (±3.28 × 10^–3^)	0.05 (±3.90 × 10^–3^)	0.07 (±4.73 × 10^–3^)	0.06 (±5.75 × 10^–3^)	0.05 (±3.99 × 10^–3^)	0.06 (±5.75 × 10^–3^)
	χ^2^	11.20	14.30	10.10	22.60	15.50	22.60
	R^2^	0.98	0.98	0.98	0.97	0.98	0.97
Pseudo-second-order	*q_max_* (mg g^−1^)	85.45 (±1.20)	85.90 (±1.41)	82.92 (±0.48)	85.99 (±1.64)	87.01 (±1.44)	87.16 (±1.84)
	*k*_2_ (L mg^−1^)	6.89 × 10^–4^ (±5.29 × 10^–5^)	7.19 × 10^–4^ (±6.31 × 10^–5^)	1.16 × 10^–3^ (±3.97 × 10^–5^)	8.66 × 10^–4^ (±8.82 × 10^–5^)	6.97 × 10^–4^ (±6.09 × 10^–5^)	8.02 × 10^–4^ (±8.57 × 10^–5^)
	χ^2^	5.04	6.45	1.14	8.78	6.40	8.74
	R^2^	0.99	0.99	1.00	0.99	0.99	0.99
Intraparticle diffusion	*k*_3_ (mg g^−1^ min^−1/2^)	5.50 (±0.40)	5.72 (±0.28)	4.92 (±0.36)	6.61 (±0.29)	6.28 (±0.26)	7.10 (±0.79)
	*A*	13.58 (±1.75)	13.55 (±1.25)	21.13 (±1.57)	12.47 (±1.27)	11.35 (±1.16)	10.34 (±3.49)
	R^2^	0.90	0.90	0.81	0.94	0.93	0.95
External-film diffusion	*D_f_* (m^2^ min^−1^)	1.10 × 10^–11^	1.21 × 10^–11^	1.13 × 10^–11^	1.38 × 10^–11^	1.25 × 10^–11^	1.42 × 10^–11^
	R^2^	0.99	0.98	0.99	0.99	0.98	0.99
Internal-pore diffusion	*D_p_* (m^2^ min^−1^)	1.3 × 10^–17^	1.4 × 10^–17^	1.3 × 10^–17^	1.6 × 10^–17^	1.4 × 10^–17^	1.6 × 10^–17^
	R^2^	0.98	0.97	0.98	0.98	0.97	0.98

**Table 6:** MB adsorption capacity of synthesized composites and of other composites reported in the literature.

Material	*q_e_* (mg g^–1^)	References
R	85.45	This study
RT	85.90	This study
RZT	82.92	This study
G	85.99	This study
GT	87.01	This study
GZT	87.16	This study
Magnetic graphene oxide modified zeolite	97.35	[91]
Raw zeolite	6.10	[92]
Activated lignin-chitosan composite extrudates	36.25	[93]
Poly (dopamine hydrochloride) (PDA) microspheres	90.70	[94]
TiO_2_/montmorillonite-albumin nanocomposite	18.18	[95]
Carboxymethyl cellulose/ZSM-5/ZIF-8	10.49	[96]
TiO_2_ nanoparticles	88.10	[97]
ZSM-5 zeolite	105.82	[48]
NaX zeolite	127.13	[98]
Chitosan-epichlorohydrin/zeolite	156.10	[99]
Chitin/clay microspheres	152.20	[100]
Magnetic chitosan/clay beads	82.00	[101]
Activated carbon-clay composite	178.64	[102]
KMgFe(PO_4_)_2_	22.83	[103]
Hydroxysodalite	10.82	[104]
Kaolin	21.41	[105]
Nonporous silica	91.10	[106]

## Data Availability

Data is contained within the article and Supplementary Materials.

## Supplementary Materials

The following are available online at https://www.mdpi.com/article/10.3390/nano11040898/s1, Figure S1. Comparison of the diffraction pattern of the RZT extrudate with the diffraction pattern of its individual components. Figure S2. Comparison of the diffraction pattern of the GZT extrudate with the diffraction pattern of its individual components. Table S1. 2θ values of the diffraction peaks and planes assigned to the peaks.
