# Supplementary material for: Structuring of ZnTiO3/TiO2 Adsorbents for the Removal of Methylene Blue, Using Zeolite Precursor Clays as Natural Additives
Source: Nanomaterials (Basel). 2021 Apr 1;11(4):898. doi: 10.3390/nano11040898 (PMC8067086; doi:10.3390/nano11040898)
Supplement: Supplementary file 1 [file nanomaterials-11-00898-s001.pdf]

nanomaterials

MDPI

Supplementary Materials

Structuring of $ZnTiO_3/TiO_2$ adsorbents for the removal of methylene blue, using zeolite precursor clays as natural additives

Ximena Jaramillo-Fierro [1,2,*]. Silvia González [1]. Fernando Montesdeoca-Mendoza [1] and Francesc Medina [2]

[1] Departamento de Química y Ciencias Exactas. Universidad Técnica Particular de Loja. San Cayetano Alto. 11-01-608 Loja. Ecuador; sgonzalez@utpl.edu.ec (S.G.); famontesdeoca2@utpl.edu.ec (F.M-M.)
[2] Departamento d'Enginyería Química. Universitat Rovira i Virgili. Av Països Catalans 26. 43007 Tarragona. Spain; francesc.medina@urv.cat
* Correspondence: xvjaramillo@utpl.edu.ec; Tel.: +593-7-3701444

Table S1. 2θ values of the diffraction peaks and planes assigned to the peaks

Compounds	2θ values	Planes
R-Clay	7.18°, 10.16°, 12.45°, 16.09°, 17.64°, 20.40°, 21.34°, 21.65°, 22.84°, 23.97°, 26.09°, 27.09°, 29.00°, 29.92°, 30.81°, 32.52°, 33.34°, 34.16°, 35.71°, 36.48°, 37.97°, 40.10°, 41.48°, 42.15°, 42.83°, 43.47°, 44.12°, 47.25°, 47.86°, 49.07°, 49.67°, 51.97°, 52.55°, 53.11°, 54.23°, 54.79°, 56.40°, 57.48° and 58.56°	(2 0 0), (2 2 0), (2 2 2), (4 2 0), (4 2 2), (4 4 0), (5 3 1), (6 0 0), (6 2 0), (6 2 2), (6 4 0), (6 4 2), (8 0 0), (6 4 4), (6 6 0), (8 4 0), (8 4 2), (6 6 4), (8 4 4), (10 0 0), (10 2 2), (10 4 2), (8 8 0), (10 4 4), (10 6 0), (10 6 2), (12 0 0), (10 8 0), (10 8 2), (12 4 4), (10 8 4), (12 6 4), (10 10 0), (14 2 2), (12 8 2), (14 4 2), (10 8 8), (10 10 6), and (12 10 0)
FAU zeolite	6.11°, 9.99°, 11.72°, 15.42°, 17.34°, 18.40°, 20.05°, 20.98°, 22.45°, 23.29°, 23.56°, 24.63°, 25.39°, 26.63°, 27.35°, 29.18°, 30.27°, 30.92°, 31.13°, 31.96°, 32.77°, 33.56°, 34.14°, 35.11°, 36.59°, 37.31°, 40.76°, 41.25°, 42.55°, 43.34°, 46.43°, 47.02°, 48.62°, 49.79°, 50.49°, 50.92°, 51.59°, 53.11°, 57.32°, 58.36°, 59.01° and 59.39°	(1 1 1), (2 2 0), (3 1 1), (3 3 1), (4 2 2), (5 1 1), (4 4 0), (5 3 1), (6 2 0), (5 3 3), (6 2 2), (4 4 4), (5 5 1), (6 4 2), (5 3 1), (7 3 3), (8 2 2), (1 5 7), (6 6 2), (8 4 0), (8 4 2), (6 6 4), (9 3 1), (8 4 4), (2 6 8), (10 2 2), (8 8 0), (11 3 1), (11 3 3), (8 8 4), (12 4 2), (10 8 2), (9 7 7), (13 3 3), (8 8 8), (13 5 1), (14 2 0), (11 9 3), (11 11 1), (1 9 13), (16 0 0) and (3 5 15)
Na-P1 zeolite	12.47°, 17.73°, 21.65°, 28.18°, 33.44°, 35.63°, 40.31°, 42.31°, 44.13°, 44.29°, 46.14°, 49.87°, 51.48°, 51.67°, 53.09°, 53.28°, 54.87°, 55.06°, 56.29°, 56.42°, 56.69°, 57.91°, 58.27°, 59.63°, 59.89°, 61.42°, 62.61°, 62.90°, 62.99°, 64.35°, 65.50°, 65.75°, 66.00°, 67.33°, 67.49°, 68.59° and 68.87°	(1 0 1), (2 0 0), (1 1 2), (3 0 1), (3 1 2), (0 0 4), (4 0 2), (3 3 2), (2 2 4), (4 2 2), (3 1 4), (5 1 2), (4 0 4), (4 4 0), (3 0 5), (4 3 3), (4 2 4), (6 0 0), (1 1 6), (3 2 5), (6 1 1), (2 0 6), (6 0 2), (4 1 5), (5 4 1), (6 2 2), (3 1 6), (6 1 3), (6 3 1), (4 4 4), (1 0 7), (4 3 5), (5 4 3), (6 0 4), (6 4 0), (3 3 6) and (6 3 3)
$ZnTiO_3$	23.92°, 32.79°, 35.31°, 40.45°, 48.93°, 53.44°, 56.82°, 61.79° and 63.39°	(0 1 2), (1 0 4), (1 1 0), (1 1 3), (0 2 4), (1 1 6), (0 1 8), (2 1 4) and (3 0 0)
Anatase ($TiO_{2\text{-}a}$)	25.28°, 36.95°, 37.80°, 38.58°, 48.05°, 53.89°, 55.06°, 62.12°, 62.69° and 68.76°	(1 0 1), (1 0 3), (0 0 4), (1 1 2), (2 0 0), (1 0 5), (2 1 1), (2 1 3), (2 0 4) and (1 1 6)
Rutile ($TiO_{2\text{-}r}$)	27.45°, 36.09°, 41.23°, 54.32°, 56.64° and 69.01°	(1 1 0), (1 0 1), (1 1 1), (2 1 1), (2 2 0) and (3 0 1)

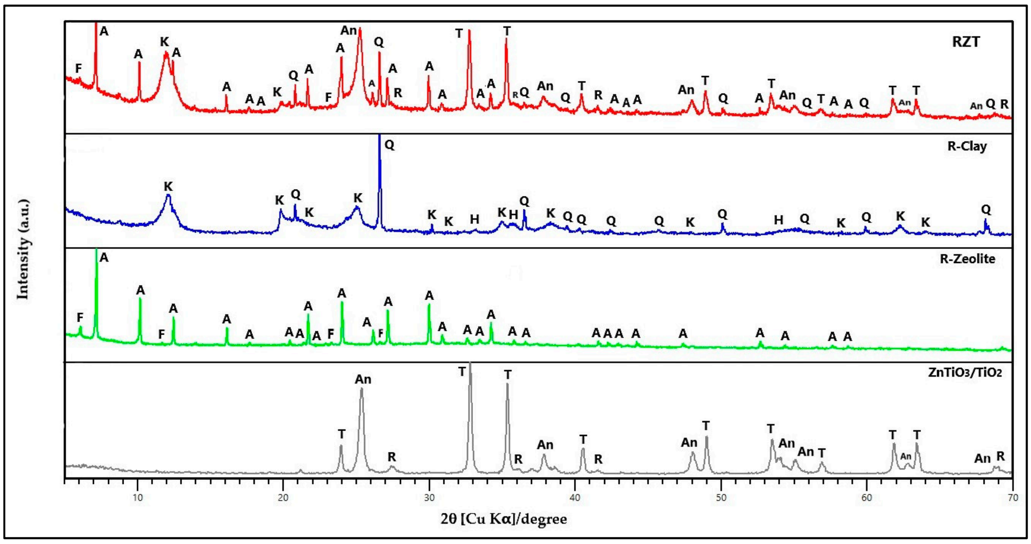

Figure S1. Comparison of the diffraction pattern of the RZT extrudate with the diffraction pattern of its individual components.

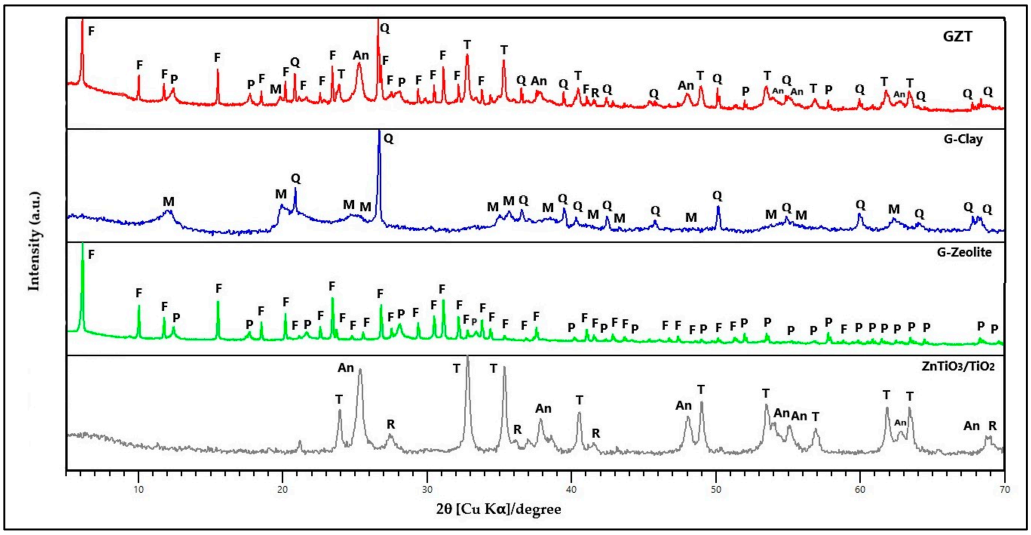

Figure S2. Comparison of the diffraction pattern of the GZT extrudate with the diffraction pattern of its individual components.
